# YAP and the Hippo pathway in cholangiocarcinoma

**DOI:** 10.1007/s00535-019-01563-z

**Published:** 2019-02-27

**Authors:** Takaaki Sugihara, Hajime Isomoto, Gregory Gores, Rory Smoot

**Affiliations:** 1 Division of Medicine and Clinical Science, Department of Multidisciplinary Internal Medicine, Tottori University Faculty of Medicine, Yonago, Tottori Japan; 2 Division of Gastroenterology and Hepatology, Mayo Clinic College of Medicine and Science, 200 First Street SW, Rochester, MN 55905 USA; 3 Division of Hepatobiliary and Pancreas Surgery, Mayo Clinic College of Medicine and Science, 200 First Street SW, Rochester, MN 55905 USA

**Keywords:** Cholangiocarcinoma, Yes-associated protein, Hippo pathway

## Abstract

Cholangiocarcinoma (CCA) has an increasing incidence and remains a difficult to treat malignancy. In a search for more effective treatment options, progress has been made in identifying molecular drivers of oncogenic signaling including IDH mutations and FGFR2 fusions. In addition, multiple investigators have identified increased activity of YAP, the effector protein of the Hippo pathway, in CCA. The Hippo pathway regulates organ size, cellular proliferation, and apoptosis via YAP, a transcriptional co-activator. Targeting of the pathway has been difficult due the lack of a dedicated cell-surface receptor. However, more recently, additional cross-regulatory pathways have been identified that are potentially targetable. In this review, we address the current treatment landscape for CCA, the Hippo pathway broadly, animal models of CCA with attention to Hippo-related models, and the current strategies for targeting YAP.

## Introduction

Cholangiocarcinoma (CCA) is a malignancy likely originating from the biliary epithelium, with an increasing incidence [1–3]. Cholangiocarcinoma can occur at multiple points along the biliary tree, and is subtyped based on anatomic criteria into intrahepatic, perihilar, and distal [2, 3]. Several risk factors have been associated with development of this tumor including hepatitis B and C infection, liver fluke infection, biliary stone disease, congenital biliary cysts, and underlying primary sclerosing cholangitis [2–4]. However, the vast majority of patients have no identifiable risk factors. While evidence continues to accrue that the subtypes of CCA have unique molecular signatures (and likely represent very different cancers), the rarity of the tumor and the paucity of treatment trials has translated into a grouping of these tumors into “biliary tract cancers” (BTC) [5, 6]. Gemcitabine and platinum-based combination chemotherapy have been defined as the standard first-line chemotherapy for unresectable or metastatic BTC [7]. Unfortunately, the benefit of first-line therapy is limited, and for those patients that progress, no standard second-line chemotherapy has yet been established. Surgical resection remains the mainstay of treatment; however, even in patients with apparently resectable disease, recurrence rates are approximately 70% and 5-year survival a mere 30% [8–10]. Adjuvant trials have examined several treatment paradigms including systemic chemotherapy and chemoradiotherapy. The results have been disappointing, with either no or very minimal improvements in overall outcomes [11–13]. SWOG S0809 is a recently reported single-arm, phase 2 trial examining outcomes in patients with BTC treated with adjuvant gemcitabine and capecitabine, followed by capecitabine plus radiotherapy. R1 patients had similar survival as those with R0 disease, and overall survival (median 35  months) was favorable suggesting some benefit from this therapy; however, no control group was included which limits conclusions from this trial [11]. In addition, two large adjuvant chemotherapy trials have been reported as abstracts and are awaiting final publication. These included the PRODIGE12-ACCORD18 trial and the BILCAP trial [12, 13]. PRODIGE12-ACCORD18 was a multicenter, randomized, phase 3 trial that evaluated adjuvant gemcitabine and oxaliplatin versus observation alone following resection of BTC. The primary endpoint in this trial was recurrence free survival, with no difference in the study groups noted. Even when subgroup analysis was completed by tumor type, no positive findings were noted [12]. The BILCAP trial was a multicenter, randomized, phase 3 trial that evaluated adjuvant capecitabine versus observation in patients with all the types of BTC. Comparing the groups, there was a notable increase in median overall survival from 36 months in the control group to 51 months in the capecitabine arm; however, this did not reach statistical significance in the intention to treat analysis. In per protocol analysis, the difference did reach significance [HR 0.75, (5% CI 0.58, 0.97; *p* = 0.028)], leading the investigators to recommend consideration of adjuvant capecitabine for all resected BTC; however, this remains controversial given the results of the intention to treat analysis [13]. Thus, there remains a critical need to develop effective therapies for the treatment of this lethal disease. Therapeutic advances will require additional insights regarding the molecular mechanisms of biliary carcinogenesis and tumor progression. Some progress has been made with the identification of isocitrate dehydrogenase (IDH) mutations and fibroblast growth factor receptor 2 (FGFR2) fusions as drivers in small subsets of intrahepatic cholangiocarcinoma tumors [5, 14–16]. Further investigation has identified the Hippo pathway as a pathway of interest, as several investigators have demonstrated activity of Hippo pathway components in CCA [17–24]. Herein, we review the Hippo pathway, regulation of its effector protein YAP, and the evidence for a role in CCA.

## The Hippo pathway

**Fig. 1:**
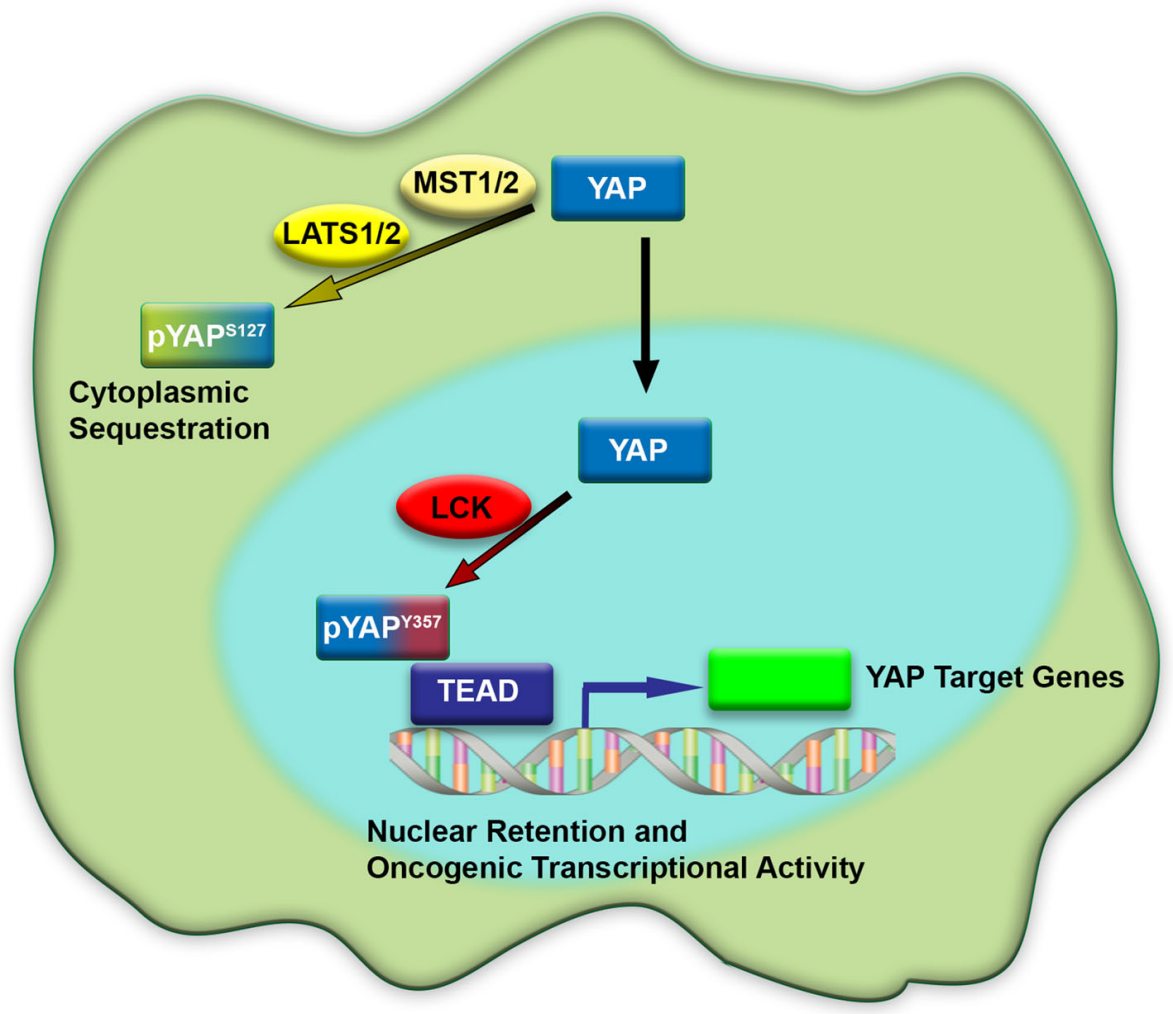
YAP regulation in cholangiocarcinoma. Schematic representation of the Hippo pathway and Src family kinase regulation of YAP in cholangiocarcinoma. *LATS* large tumor suppressor, *LCK* LCK Src family kinase, *MST* mammalian sterile 20-like kinase, *TEAD* TEA-domain protein

The Hippo pathway is important in control of organ size and consists of a series of serine/threonine kinases [mammalian sterile-like 20 (MST1/2), large tumor suppressor (LATS1/2)] and scaffolding proteins [salvador (SAV), mps one binder (MOB1)] which regulate the subcellular localization and activity of the effector proteins [yes-associated protein (YAP), transcriptional co-activator with a PDZ-binding domain (TAZ)], which function as transcriptional co-activators [25] (Fig. 1). Interestingly, the Hippo pathway does not have a dedicated cell-surface receptor and consequently is regulated via cross-talk with additional signaling pathways [23, 26, 27]. Furthermore, mutations in Hippo pathway components themselves are uncommon in human CCA, placing additional emphasis on understanding post-translational regulatory mechanisms in driving Hippo/YAP activity in CCA [28].

Components of the Hippo pathway were first discovered via mutational screens in *Drosophila* [29–32]. Phenotypically, flies demonstrated massive overgrowth of various epithelial structures. The involved genes were determined to be regulatory serine/threonine kinases corresponding to the human proteins MST1/2 and LATS1/2 [31]. These kinases were found to regulate YAP via a serine phosphorylation cascade culminating in phosphorylation of serine 127 on YAP. Phosphorylation of YAP at this serine residue is associated with binding of YAP to 14–3–3 proteins, functionally sequestering YAP in the cytoplasm and limiting its activity as a transcriptional co-activator [33]. Accordingly, the canonical regulation of the pathway is such that when the Hippo pathway is “active”, YAP in restrained; and thus, when the Hippo pathway is “inactive”, YAP is free to bind to transcription factors and enhance transcription. YAP has been demonstrated to bind to multiple transcription factors; however, it most commonly associates with the TEA-domain (TEAD) transcription factors [23, 34, 35]. Multiple YAP-TEAD target genes have been identified, with connective tissue growth factor (CTGF) and cysteine rich angiogenic inducer 61 (CYR61) representing two of the more commonly assayed as a readout of YAP activity [36, 37]. Other YAP target genes of note that have been previously identified in various cell types are CyclinD1, BCL-XL, and BIRC5 [34, 38]. In CCA, we have identified FGFR1,-2,-4, PDGF-B, and MCL-1 as YAP target genes [22–24]. Others have demonstrated that ANKRD1 and the pro-angiogenic MFAP5 are also a YAP target genes in CCA [18].

In addition to the canonical regulatory serine phosphorylation, other regulatory post-translational modifications have been identified, including tyrosine phosphorylation [23, 24, 39]. Phosphorylation of the YAP tyrosine 357 residue has been demonstrated in the setting of both cancer and inflammation. In an intestinal inflammation model, IL-6-mediated activation of Src family kinases (SFK) was found to culminate in YAP activation via tyrosine phosphorylation [39]. The same group that identified IL-6-mediated activation in inflammation, subsequently identified activation of YAP via an IL-6 mediated mechanism in colon cancer after APC gene loss [40]. Specific to CCA, our group identified SFK activation and subsequent YAP activation downstream of receptor tyrosine kinase (platelet-derived growth factor) activation [23]. Furthermore, we identified LCK as the SFK member most responsible for YAP phosphorylation in CCA, and that tyrosine phosphorylation could regulate YAP subcellular localization and activity independent of the canonical serine regulatory mechanisms [24]. This last observation was an important distinction, as SFK activity has been shown to be able to regulate the activity of the serine kinase LATS. Such that increased SFK activity can decrease the activity of LATS, leading to YAP that is not restrained by serine phosphorylation [41]. In our CCA models, we did not observe an effect of SFK inhibition on LATS activity, but rather only directly on the tyrosine phosphorylation status of YAP [24]. Our observations led us to label the tyrosine phosphorylation as a nuclear retention signal for YAP. The concept of tyrosine phosphorylation as a nuclear retention signal for YAP was supported by recent work identifying SRC as a direct regulator of YAP export from the nucleus by regulating binding to exportin1 [42].

A variety of extra-cellular signals have been shown to regulate YAP subcellular localization/activity, even though no dedicated receptor exists for the pathway. Initial work in this area identified lysophosphatidic acid (LPA) and serum signaling, via G-protein linked receptors, as regulators of the Hippo pathway [27]. These observations were extended to mitogenic signaling via epidermal growth factor (EGF) [26]. Evaluation of EGF-mediated inhibition of the Hippo pathway in a mammary cell line, identified phosphoinositide 3-kinase (PI3K) and phosphoinositide-dependent kinase-1 as important downstream mediators of this receptor-mediated regulation, and suggested that PI3K activation may be a conserved mechanism of Hippo inhibition via multiple mitogenic signals [26]. In CCA, we have identified activation of YAP by platelet-derived growth factor (PDGF) and fibroblast growth factor (FGF) [22, 23]. In the case of PDGF, the activation of YAP appears to be mediated directly through activity of Src family kinases on YAP tyrosine residues, in contrast to inhibition of the Hippo pathway [23]. In an inflammatory model, interleukin-6 has been demonstrated to activate YAP directly in a similar fashion via the gp130 receptor activating SFKs [39]. Non-receptor-mediated regulators of the Hippo pathway, and YAP activity, include cell–cell contact, and mechano-transduction [43–45]. Mechano-transduction is of interest given the desmoplastic nature of CCA and the fact that it has previously been shown to be important in human liver cancer. Specifically, the extra-cellular matrix (ECM) proteoglycan Agrin was demonstrated to relay ECM stiffness signals through focal adhesion kinases, inhibiting the Hippo pathway, activating YAP, and driving hepatocellular oncogenesis [43]. ECM signaling and YAP activation in CCA has yet to be explored.

## YAP/Hippo and Human CCA

Several groups have evaluated the expression levels and subcellular localization of YAP in specimens of human cholangiocarcinoma [17–21]. A variety of thresholds have been utilized to define YAP expression and localization, and the subtype of cholangiocarcinoma has not been clearly defined; however, a majority of CCA specimens have demonstrated YAP staining that is nuclear localized (if localization was evaluated). Furthermore, YAP levels were correlated with prognosis in several cohorts. Sugimachi et al. evaluated YAP expression levels in 88 intrahepatic cholangiocarcinoma specimens and found the lowest reported level of YAP overexpression at approximately 32% of the examined specimens, although this was specific for YAP overexpression and not total YAP expression (or localization). Those patients with tumors demonstrating YAP overexpression had significantly decreased survival [20]. Similarly, Wu et al. examined YAP staining in 122 cholangiocarcinoma specimens and found 67% with YAP expression which correlated with worse outcomes [21]. Other groups have observed a higher rate of YAP positivity with nuclear localization. For example, Marti et al. reported on 107 CCA specimens and observed a YAP positivity rate of 88% with 85% of those tumors demonstrating nuclear localization of YAP. Importantly, this group evaluated SOX9 and CK19 staining and included only those specimens positive for both [18]. Comparably Pei et al. reported on evaluation of 90 specimens in which 94% were found to be YAP positive with the majority being nuclear localized, and Li. et al. reported on a smaller cohort of 16 CCA specimens, but 98% demonstrated significant nuclear YAP staining [19]. This collection of studies further supports a role of YAP in CCA biology; however, we caution that expression levels alone are unlikely to truly represent YAP oncogenic activity, and additional studies will likely need to evaluate YAP cognate target gene expression as a surrogate of activity as phosphorylation status is difficult to assess utilizing standard immunohistochemical approaches.

The drivers of YAP activation in CCA have yet to be fully defined. Our group has previously reported on PDGF and FGF modulation of YAP/Hippo activity in human CCA cell lines, and has demonstrated that upregulated SFK activity could “activate” YAP; however, whether this is recapitulated in vivo is incompletely understood. Importantly, genetic alterations of YAP and/or other Hippo pathway components appear to be an uncommon event in human CCA and are not likely to represent significant drivers of tumorigenesis. The recently reported TCGA analysis of CCA specimens demonstrated only 5% of specimens with a mutation in Salvador, and 3% with an NF2 mutation. This cohort was made up of 38 specimens with the majority (89%) being from North America, and the majority (84%) being intrahepatic CCA; however, there are no data to indicate that different cohorts would have an increased frequency of mutations [28].

The downstream consequences of YAP activation in CCA have been explored by several groups with upregulation of YAP expression associated with increased cancer cell growth, xenograft tumor growth, and resistance to treatment [18, 19, 22–24]. Marti et al., identified both down regulation of the pro-death molecule TRAIL in YAP overexpressing CCA cell lines, but also an upregulation of the pro-angiogenic protein MFAP5 [18]. Importantly, these changes were dependent on TEAD transcription factor binding, as YAP bearing an S94A mutation (which limits the ability to bind TEAD proteins) did not induce these changes. Pei et al. further demonstrated that YAP upregulation was associated with epithelial to mesenchymal (EMT) transition and could increase the expression of gankyrin which was subsequently noted to upregulate YAP via an IL-6-mediated mechanism [19]. This concept of a feed-forward loop of YAP activation (or Hippo inhibition) via YAP-driven transcriptional targets was also reported with both PDGF and FGF signaling in CCA by our group [22, 23].

## YAP/Hippo and animal models of CCA (Table 1)

Multiple murine models of CCA have been developed and are well summarized in a recent review; however, the majority of these are not specific to YAP/Hippo aberrations [46]. Initial studies evaluating the consequence of genetic deletion of Hippo pathway components in murine models demonstrated high levels of mortality, and subsequent conditional knockouts demonstrated tissue overgrowth (especially in the liver) and eventual tumor formation. For example, both liver specific MOB1a/1b double knockout mice and MST1/2 conditional knockout mice demonstrated liver tumors that either had mixed HCC and CCA components or predominantly HCC tumors with a smaller frequency of CCA tumors [47, 48]. Given the length of time to develop tumors and the mixed phenotypes of the tumors neither of these genetic models represents a functionally useful cancer-specific model.

**Table 1:** YAP/Hippo murine models of cholangiocarcinoma

Model	Methods	Timeframe (weeks)	Histology
S127A-YAP/myr-AKT transposon [50]	Biliary instillation of SB based transposons surgically	6–8	Intrahepatic cholangiocarcinoma
TetO-YAP1 [49]	Transgenic S127A-YAP under Tet control	10–12	Mixed HCC and cholangiocarcinoma
Mob1a^−/−^/Mob1b^−/−^ [47]	Double knockout	20–40	Mixed HCC and cholangiocarcinoma
Mst1^−/−^/Mst2^c/−^ [48]	Tamoxifen-inducible double knockout	24	Mainly HCC with some cholangiocarcinoma

A doxycycline-inducible activated YAP (S127A-YAP) has also been inserted downstream of the collagen 1a1 locus and crossed with mice expressing the tretracycline transactivator on the liver activator protein promoter [49]. These animals demonstrate liver hypertrophy and eventual tumor formation, although the histology is generally mixed HCC/cholangiocarcinoma as well.

Our group has reported on the development and validation of a YAP-driven, transposon-mediated, murine model of CCA [50]. In this model sleeping beauty transposons containing an activated YAP (S127A-YAP) as well as myristolated AKT (myr-AKT) are injected into the biliary tree of a mouse following surgical exposure. The animals are treated with IL-33 intraperitoneally for 3 days to facilitate mitogenic growth of the cholangiocytes, likely opening up the chromatin and facilitating transposon integration. In this model, greater than 70% of animals develop tumors that are histologically and immunophenotypically consistent with intrahepatic cholangiocarcinomas in 6–8 weeks. Interestingly, both activated YAP and myr-AKT are necessary for tumor formation, and omission of IL-33, a potent biliary mitogen, significantly reduces the efficiency of tumor formation from ~ 70% down to ~ 20%. Tumor formation in this model appears to require IL-6, as this can be substituted for IL-33, and tumor formation is completely eliminated in IL-6 knockout mice [50].

## Targeting YAP/Hippo (Fig. 2)

Targeting the core Hippo pathway in cancer has been difficult given the negative regulatory function of the core kinases. As such, therapeutic approaches have focused on targeting YAP-TEAD interactions or other cross-regulatory pathways. One such approach has been to utilize the benzoporphyrin, verteporfin. This compound is utilized currently clinically as a photosensitizer for photodynamic therapy in macular degeneration. It has been demonstrated to interrupt the YAP–TEAD4 interaction and has demonstrated some efficacy in models of various tumors [51, 52].

**Fig. 2:**
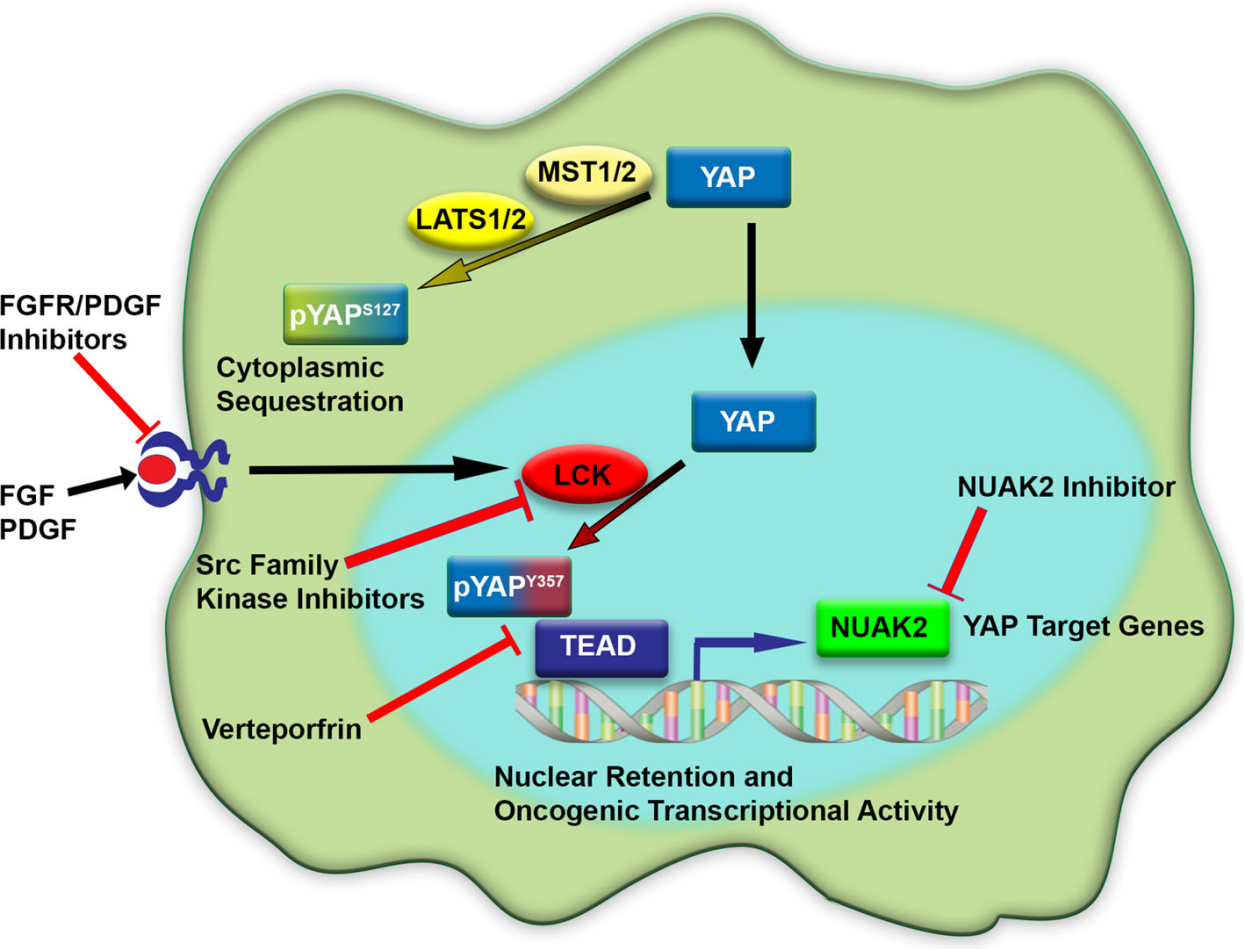
Therapeutic targeting of YAP in cholangiocarcinoma. Schematic representation of YAP targeting strategies in cholangiocarcinoma. *FGF* fibroblast growth factor, *LATS* large tumor suppressor, *LCK* LCK Src family kinase, *MST* mammalian sterile 20-like kinase, *PDGF* platelet-derived growth factor; TEAD, TEA-domain protein

An additional therapeutic approach that may target YAP/Hippo and currently is being explored in clinical trials in cholangiocarcnoma is FGFR inhibition. Trials assessing this approach arose out of the observation that approximately 15% of intrahepatic cholangiocarcinomas express an FGFR2 fusion protein [14, 15]. The role of YAP in these tumors is incompletely understood; however, we previously demonstrated an FGF–YAP–FGFR autocrine loop that drove oncogenic signaling in multiple CCA models. Downregulation of FGF signaling in these models, utilizing a small molecule inhibitor, disrupted this autocrine loop, and inhibited YAP activity [22]. These preclinical studies suggest that YAP activation/localization may serve as a biomarker for identifying patients most likely to benefit from FGF-targeted therapy and that this treatment strategy may be efficacious even in some patients without an FGFR2 fusion protein. The outcomes of the on-going clinical trials in CCA, and the correlative studies, will hopefully shed some light on these questions.

In the preclinical setting, additional kinase inhibitors have demonstrated modest efficacy. Specifically, downregulating PDGF signaling utilizing a small molecule inhibitor, crenolanib, was associated with downregulation of SFK activity, YAP tyrosine phosphorylation, and CCA viability in CCA cell lines [23]. In addition, the SFK inhibitor dasatinib has demonstrated significant downregulation of YAP tyrosine phosphorylation and CCA viability both in vitro and in patient-derived xenograft models of CCA [24].

Recent work has identified another potential target in a genetic liver cancer model driven by activate (S127A) YAP which was also found to be present in CCA cell lines. Yuan et al. utilized a ChIP-Seq/bioinformatics approach to identify YAP-driven targets with enzymatic activity that could be potentially targeted in hepatocytes bearing the S127A mutant YAP as well as the HuCCT1 CCA cell line. The investigators identified NUAK2 as a YAP target gene and utilized a semi-specific small molecule inhibitor to demonstrate that inhibition of this enzyme decreased the growth rate of liver cancer cells in vitro as well as the growth rate of HuCCT1 cell line xenografts in mice. Weights of the mice were similar at the end of treatment, suggesting limited toxicity; however, further confirmatory studies will be required [37].

Finally, other therapeutic approaches being explored include utilizing siRNA techniques as well as peptide inhibitors meant to disrupt the interaction between YAP and TEAD [53]. These approaches have demonstrated some efficacy in experimental models, however, continue to have significant challenges with translation due to difficulties with delivery in vivo.

## Conclusion

Evidence continues to accumulate demonstrating the importance of the Hippo pathway and its effector protein YAP in human cancers broadly, and in cholangiocarcinoma more specifically. The frequency of “active” YAP in these tumors is as striking as the infrequency of somatic mutations affecting the Hippo pathway components. Based on this, molecular explorations, and therapeutic approaches continue to explore regulatory cross-talk from other signaling pathways. Reproducible animal models now exist, and will aid in delineating the molecular events unpinning these tumors as well as the evaluation of new targeted therapeutic approaches.
